# Myiasis of Maxilla: Report of a Case

**DOI:** 10.5005/jp-journals-10005-1119

**Published:** 2011-04-15

**Authors:** Syed Sayeed Ahmed, Wakil Ahmed, Afshan Bey

**Affiliations:** 1 Associate Professor, Department of Oral and Maxillofacial Surgery, Dr Ziauddin Ahmed Dental College, Aligarh Muslim University Aligarh, Uttar Pradesh, India; 2 Dental Surgeon, Department of Oral and Maxillofacial Surgery, Dr Ziauddin Ahmed Dental College, Aligarh Muslim University Aligarh, Uttar Pradesh, India; 3 Professor, Department of Periodontics and Community Dentistry, Dr Ziauddin Ahmed Dental College, Aligarh Muslim University Aligarh, Uttar Pradesh, India

**Keywords:** Myiasis, Musca nebulo, Ivermectin.

## Abstract

Oral myiasis is a disease caused by larvae of housefly and associated with poor oral hygiene, mouth breathing and incompetent lips. The disease is characterized by presence of maggots in affected parts of the body and is usually seen in tropical countries, although cases have been reported from other parts of the world. There are various methods for treating this disease. In this case report, a combination of treatment modalities has been favored.

## INTRODUCTION

Myiasis is defined as invasion of the organs and tissues of human or vertebrate animals with dipterous larvae.^1^ These larvae invade to human tissues or cavities,^2^ where they feed and cause debilitating myiases.^3^

Myiasis is rare disease in human and seen individuals with poor oral hygiene, malnutrition, senility and suppuration, or malignancies. Its incidence is worldwide, and white and black, both races are affected. The disease affects those areas of body which are accessible for egg laying of flies and the condition favors the development of larvae which are seen as maggots.

This paper is a case report based on a case of oral myiasis seen in anterior region of maxilla.

## CASE REPORT

**Fig. 1:**
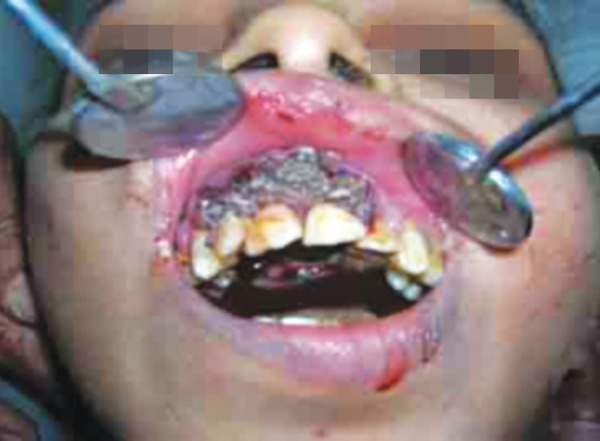
Large number of maggots

A 16-year-old male, malnourished patient suffering with hypotonic cerebral palsy/poliomyelitis, attended our department with chief complaint of acute dental pain in maxillary teeth. Intraoral examination revealed the poor oral hygiene with severe halitosis and swelling in premaxillary region. The patient was a mouth breather, had type II malocclusion, and incompetent upper and lower lips. Maxillary anterior teeth were tender. Further, a large number of moving maggots were present in the pocket between labial gingival and labial cortical plate (Fig. 1). On the basis of clinical examination and history and clinical presentation, the disease was diagnosed as oral myiasis.

The mobile maggots were first removed using clinical forceps. After their removal, more maggots were seen in deeper parts and it was to remove them by forceps, so other method was used. A cotton swab impregnated with turpentine oil was then placed at orifice. Turpentine oil acts as an irritant to the larvae and they move away from their hide. After 5 minutes, more maggots were appeared in that area from where the maggots were removed earlier. They were again removed by forceps and the process was repeated. The entomologist identified them as larva of housefly, musca nebulo. After identification of larvae, 6 mg ivermectin was given orally and repeated after 24. On next day, the remaining series of larvae were immobile.

The affected area was irrigated with hydrogen peroxide and povidine iodine solution in the consecutive days until none maggot was clinically seen. Signs of clinical healing were also evident. Oral mucosa appeared returning to normal state and attaching to bone.

## DISCUSSION

The term myiasis (from Greek: myi = mosca) refers to infestation of living tissues of animals or humans^4,5^ by larvae of diptera,^6^ chrysomya bezziana,^7^ oestrus ovis,^8^ wohlfahrtia magnifica,^9^ etc.

The predisposing factors of myiasis are poor hygiene, senility, alcoholism and neurologic disorders. For oral myiasis, mouth breathing, poor oral hygiene are important and neurological disorder may or may not be associated. In Indian continent, the disease is caused by housefly *Musca nebulo.* In a neurologically compromised mouth breather patients or with incompetent lips, patient is unable to take care of himself. Thus, flies sit on exposed parts and lay eggs which develop into larvae. They are photobhobic, thus tend to hide deep in soft tissues, and feed on tissues of the host. The larvae release toxins which destroys the host tissues. Surrounding bacteria decompose the tissues and larvae feed on them. The infected tissue releases a foul smelling discharge which produces severe halitosis. In chronic cases, they cause severe bone and soft tissue destruction that may lead to life-threatening hemorrhage also.^4^ However, the extent of damage depends upon the duration of infestation, more delayed the diagnosis and greater the number of larvae, the greater the damage to tissues.^5^

The treatment of oral myiasia has been reported as (i) mechanical removal, (ii) local application of asphyxiating agents and (iii) medicinal treatment by systemic ivermec-tin.^4^ Considering the indication of ivermectin in parasitosis, we used it in addition to rest of the two methods and there was no recurrence in the 2 years of follow-up. Addition of ivermectin in the treatments ensures death of present and developing larvae. In our opinion, ivermectin should always be used with other measures. It is safe and effective and and makes the treatment complete for myiasis.
